# Guidelines for setting cut-off scores in AUC (AUC-GUIDE): balancing sensitivity, specificity, and purpose

**DOI:** 10.3389/frma.2026.1808675

**Published:** 2026-06-05

**Authors:** Mohamad Adam Bujang

**Affiliations:** Clinical Research Centre, Institute For Clinical Research, National Institutes of Health Ministry of Health Malaysia, Sarawak General Hospital, Sarawak, Malaysia

**Keywords:** AUC, diagnostic, screening, sensitivity, specificity

## Abstract

Determining ideal cut-off scores is a critical step in screening and diagnostic test evaluation. While the Area Under the Curve (AUC) with Receiver Operating Characteristic (ROC) curve are frequently reported, these do not directly indicate the best threshold for decision-making. In addition, pursuing an optimal solution might not always be practical. This paper presents a practical guideline for selecting cut-off scores based on AUC values in conjunction with sensitivity, specificity, and intended scientific use. The study outline recommended strategies for different AUC ranges, incorporating realistic considerations and scientific priorities. The framework offers flexibility, including when to prioritize sensitivity, specificity, or balanced performance via Youden's Index and other techniques. This guideline aims to support researchers and clinicians in making evidence-based, transparent, and context-sensitive decisions when interpreting screening or diagnostic accuracy results.

## 1 Introduction

Screening and diagnostic research have gained increasing popularity in recent years, particularly with the rapid advancement of artificial intelligence (AI) and machine learning (Vaish et al., 2021; Nassif et al., 2022; Kumar et al., 2023). However, a notable gap persists pertaining to the lack of standardized guidance for selecting cut-off scores based on AUC values. In response, scholars have proposed various techniques for determining optimal cut-offs (Youden, 1950; Metz, 1978; Greiner et al., 2000; Pepe, 2003; Fluss et al., 2005). While the formulas used to determine these cut-offs may be mathematically valid, their application can be questionable if not supported by strong justifications.

### 1.1 Sensitivity, specificity, ROC, AUC, and Youden Index

Sensitivity is a fundamental measure used to evaluate the performance of a diagnostic test, screening instrument, or prediction model. It refers to the ability of a test to correctly identify individuals who truly have the condition of interest and is therefore also known as the true positive rate. Sensitivity is calculated as the proportion of actual positive cases that are correctly classified by the test. A highly sensitive test minimizes false-negative results and is particularly important in situations where failing to detect a disease may have serious clinical consequences, such as delayed treatment, disease progression, or missed opportunities for intervention. Consequently, diagnostic and screening programs designed for severe or potentially life-threatening conditions often prioritize high sensitivity to ensure that affected individuals are identified at an early stage.

Specificity is another essential measure of diagnostic performance and reflects the ability of a test to correctly identify individuals who do not have the condition of interest. Also referred to as the true negative rate, specificity is calculated as the proportion of actual negative cases that are accurately classified as disease-free. A highly specific test minimizes false-positive results and reduces the likelihood that healthy individuals will be incorrectly identified as having a disease. High specificity becomes particularly important when positive screening results trigger invasive, costly, or psychologically burdensome follow-up procedures. In low-prevalence populations, even a small false-positive rate may generate a substantial number of false-positive cases, potentially reducing the practical usefulness and positive predictive value of a screening program. Therefore, achieving an appropriate balance between sensitivity and specificity remains a critical consideration in the development and evaluation of diagnostic tools.

Receiver Operating Characteristic (ROC) analysis is a widely used statistical approach for evaluating the diagnostic performance of a test, screening instrument, or prediction model that aims to distinguish between two conditions, such as the presence or absence of a disease. The ROC curve provides a visual representation of this performance by plotting sensitivity, also known as the true positive rate, against one minus specificity, the false positive rate, across all possible cut-off values of the test. Each point on the curve corresponds to a different threshold, illustrating the trade-off between sensitivity and specificity. A ROC curve that bows toward the upper left corner indicates strong discriminative ability, whereas a curve that lies close to the diagonal line suggests that the test performs no better than chance.

The overall discriminative ability of a diagnostic test is commonly quantified using the Area Under the Curve (AUC). The AUC condenses the information from the entire ROC curve into a single summary measure ranging from 0.5 to 1.0. An AUC value of 0.5 reflects no discriminative power, equivalent to random classification, while values closer to 1.0 indicate superior diagnostic accuracy. However, although AUC is generally considered independent of disease prevalence, its practical application has limitations because clinical interpretation and decision-making often occur in populations with varying prevalence rates. Differences in prevalence can substantially influence measures such as positive and negative predictive values and affect the practical utility of selected cut-off scores (Eisenberg, 1995; Gilat and Linn, 2018). Conceptually, the AUC represents the probability that a randomly selected individual with the condition of interest will have a higher test score than a randomly selected individual without the condition. Because it is independent of any specific cut-off point and unaffected by disease prevalence, the AUC has become a standard metric for comparing the performance of diagnostic tools and predictive models in medical research.

While the ROC curve and AUC characterize the diagnostic performance of a test across a range of sensitivity and specificity values, clinical and research applications often require the selection of a specific cut-off score to classify individuals. The Youden Index is a commonly used criterion for identifying this optimal threshold (Youden, 1950). Defined as the sum of sensitivity and specificity minus one, the Youden Index ranges from 0 to 1, with higher values indicating better test performance. The cut-off point that maximizes the Youden Index is considered optimal because it achieves the best balance between sensitivity and specificity. This approach is particularly appropriate when the consequences of false-positive and false-negative classifications are assumed to be equally important.

Together, ROC analysis, AUC, and the Youden Index provide a comprehensive framework for evaluating and applying diagnostic tests. The ROC curve offers a graphical assessment of performance across all thresholds, the AUC summarizes the test's overall ability to discriminate between groups, and the Youden Index facilitates the practical selection of an optimal cut-off point. When used appropriately, these methods enhance the interpretability and clinical relevance of diagnostic and screening instruments, making them indispensable tools in modern medical and health research.

## 2 Literature review

This narrative review selected 16 studies found from literature discussing on setting AUC cut-off from the pioneer Youden index since 1950s. Table 1 summarized findings from literature regarding recommended strategy in deciding a cut-off based on AUC analysis.

**Table 1:** Summary of findings from literature regarding development and discussion on AUC.

No.	Article	Descriptions
1	(Youden, 1950)	Introduced Youden index.
2	(Metz, 1978)	Framed the Youden Index geometrically as the maximum vertical distance between the ROC curve and the diagonal reference line, strengthening its statistical interpretation.
3	(Greiner et al., 2000)	Examined cutoff selection strategies emphasizing clinical priorities, indirectly challenging equal-weight assumptions of the Youden Index.
4	(Pepe, 2003)	Critiqued the Youden Index for ignoring clinical consequences and positioned it as a statistical benchmark rather than a utility-based decision rule.
5	(Fluss et al., 2005)	Proposed methods to estimate confidence intervals for the Youden Index and its optimal cutoff, addressing uncertainty in diagnostic thresholds.
6	(Perkins and Schisterman, 2006)	Extended the original index by introducing weights to reflect unequal importance of sensitivity and specificity in different clinical contexts.
7	(Vickers and Elkin, 2006)	Demonstrated limitations of Youden-based cutoffs and introduced net benefit–based approaches as clinically meaningful alternatives.
8	(Schisterman et al., 2008)	Incorporated disease prevalence into cutoff selection, aligning the index more closely with population-level diagnostic performance.
9	(Liu, 2012)	Introduced a concordance probability evaluating the classification accuracy of a dichotomized measure is defined as an objective function of the possible cut point.
10	(López-Ratón et al., 2014)	Developed a code using R software to determine optimal cut-off score.
11	(Rota and Antolini, 2014)	Categorization is often needed for clinical decision making when dealing with diagnostic (prognostic) biomarkers and a binary outcome (true disease status).
12	(Bujang and Adnan, 2016)	To determine whether (or not) a specific tool or instrument can be used as a screening tool; then researchers will have to ensure that it has a sufficiently-high degree of sensitivity, but a lower degree of specificity can be tolerated.
13	(Habibzadeh et al., 2016)	The cut-off value is not universal and should be determined for each region and for each disease condition.
14	(Unal, 2017)	This method defines the optimal cut-point value as the value whose sensitivity and specificity are the closest to the value of the area under the ROC curve and the absolute value of the difference between the sensitivity and specificity values is minimum.
15	(Hajian-Tilaki, 2018)	Recommend Euclidean index, or product index.
16	(Bujang, 2023)	For a diagnostic purpose, the researcher will usually aim to have an excellent level of both sensitivity and specificity. Meanwhile, for a screening purpose, the researcher will usually aim to have achieved an excellent level of either sensitivity or specificity, but not both.

## 3 Methodological gap

Studies might report the AUC as a summary measure of performance without clearly articulating how this information translates into meaningful scientific decisions (Lobo et al., 2008; Kwegyir-Aggrey et al., 2023). This lack of clarity often leads to arbitrary or inconsistent selection of thresholds, which can compromise the utility and reliability of diagnostic tests (Verbakel et al., 2020). By condensing the entire ROC curve into a single number, researchers risk overlooking the rationale of performance at different cut-off points. For example, two tests with similar AUC values may have very different sensitivities and specificities at clinically relevant thresholds, yet this distinction is obscured if only the AUC is reported.

Furthermore, established methods for cut-off determination, such as Youden's Index even for other techniques, are sometimes applied incorrectly without proper consideration of the scientific intent or the risks associated with misclassification (Hassanzad and Hajian-Tilaki, 2024). Although Youden's Index provides a mathematically convenient method for identifying an “optimal” threshold, it does not inherently account for the clinical consequences of false-positive or false-negative classifications. Applying it without considering the scientific intent, prevalence of the condition, or the relative costs of misclassification can compromise the reliability of a diagnostic test and reduce its practical relevance in real-world settings.

Given these issues, there is a clear need for a practical and interpretable framework that links AUC-based test performance with cut-off decision-making, tailored to the specific purpose Addressing this methodological gap would improve transparency, standardization, and scientific relevance in the interpretation of ROC analysis results. Guidelines such as the STARD checklist help researchers report study design and outcomes in scientific publications (Bossuyt et al., 2015; Sounderajah et al., 2021). However, more detailed guidance is required to determine cut-offs based on AUC, especially when accounting for diverse clinical and research scenarios.

## 4 A way forward: aim

Despite the widespread use of receiver operating characteristic (ROC) analysis and the area under the curve (AUC) as indicators of test performance, the process of selecting an optimal cut-off score often remains arbitrary or insufficiently justified. As shown in Table 1, numerous scholars have proposed different formulas for identifying an ideal cut-off score, while others emphasize the need to clearly explain the strategy used for cut-off selection. However, the practical approaches adopted by researchers vary considerably across studies.

In response to this gap, the present paper introduces a structured guideline for determining cut-off scores based on AUC categories and the intended purpose of the test. This framework is intended to complement existing recommendations and enhance methodological rigor. It should be emphasized that this paper does not propose a new statistical technique for cut-off selection; rather, it offers a systematic and transparent approach to guide how cut-off scores should be determined.

## 5 Proposed guideline for cut-off score selection based on AUC

Therefore, the study proposes the following evidence-informed strategies for determining cut-off scores, categorized by AUC range. Five categories of cut-off values are introduced, accompanied by proposed strategies and scientific justifications as described in Table 2.

**Table 2:** Recommended cut-off for AUC, strategy and justifications.

AUC	Recommended strategy	Rationale
<0.5	Do not determine a cut-off.	Model performs worse than chance. Improve the test and/or redefined subjects' characteristics.
0.5 – <0.7	Use cut-off achieving ≥80% sensitivity for screening/triage.	Suitable for settings prioritizing screening strategy and minimizing false negatives. Selecting the cut-off should depend on the resources and operational constraints.
0.7 – <0.8	Recommend optimal cut-off (i.e., using Youden's Index, etc.) or emphasize sensitivity/specificity (i.e., ≥90.0%) depending on test purpose.	Moderate accuracy. Allows trade-offs between false positives and negatives. Selecting the cut-off should depend on the resources and operational constraints.
0.8 – <0.9	Recommend prioritize to use optimal cut-off (i.e., using Youden's Index, etc.) or aim for 100% sensitivity/specificity if scientific justified and feasible.	Strong performance. Enables confident decisions for rule-in or rule-out strategies. Selecting the cut-off should depend on the resources and operational constraints.
≥0.9	Excellent discrimination. Prioritize to use 100% sensitivity/specificity if attainable, or apply optimal cut-off (i.e., using Youden's Index, etc.).	Highly accurate. Appropriate for diagnostic confirmation and critical decision-making.

## 6 Discussion

The guideline presented in this paper addresses a methodological gap in screening and diagnostic research pertaining in determining optimal cut-off scores based on both AUC and test purpose. While the AUC provides a summary measure of a test's discriminatory ability, its scientific utility depends on meaningful interpretation within specific contexts. The current landscape lacks consensus on how to make these decisions transparently and consistently, often resulting in misapplication of statistical indices or neglect of sensitivity–specificity trade-offs.

Table 2 provides a guideline for setting cut-off scores rather than proposing a new calculation to obtain an optimal score. This guideline is useful in directing researchers on the appropriate actions to take based on the observed AUC. In other statistical tests, such as the independent-samples *t*-test and correlation analysis, effect sizes can be calculated to represent the magnitude of an association (Cohen, 1992; Bujang, 2026). In this context, statistics such as sensitivity and specificity can be regarded as effect size measures that reflect the magnitude of diagnostic accuracy.

In the literature, it is rare for researchers to set cut-offs when AUC <0.5, as such a coefficient indicates that the test does not reflect the gold standard at all. Studies of this nature are unlikely to be published due to their low or poor scientific significance, and results are often statistically insignificant (*p* > 0.05). However, a test begins to show potential when the AUC exceeds 0.5. Before deciding on a cut-off, researchers should first ensure the result is statistically significant, which underscores the importance of careful sample size planning (Bujang and Adnan, 2016; Bujang, 2023).

Moderate accuracy, such as an AUC between 0.5 and <0.7, is common in the literature, particularly in exploratory work and validation studies (Tan et al., 2013; Md Sani et al., 2017; Bujang et al., 2019; Samsudin et al., 2022). In such cases, the recommendation is to focus on a screening strategy rather than pursuing an “optimal” solution, which may be irrelevant or impractical. Researchers may choose a sensitivity of 80% or higher, depending on available resources and operational constraints. In other words, when reporting a moderate AUC, the emphasis should be on screening rather than balancing sensitivity and specificity.

An AUC between 0.7 and <0.8 may be considered moderate-to-good. Here, researchers could prioritize high sensitivity or specificity (e.g., >90%) depending on the test's purpose, or determine the optimal cut-off using Youden's Index or other techniques. However, given the relatively modest discrimination, this cut-off may not be suitable for critical conditions where lives are at stake (Md Sani et al., 2017; Bujang et al., 2018, 2019; Samsudin et al., 2022). A more desirable scenario is when the AUC falls between 0.8 and <0.9. Such results indicate strong performance, with discrimination approaching that of the gold standard (Senthil et al., 2015; Tiong et al., 2018). In this case, the test enables confident rule-in or rule-out strategies, and researchers can pursue an optimal solution (i.e., Youden index or other techniques) as a high priority method or target 100% either sensitivity or specificity.

An AUC ≥0.9 indicates excellent discrimination, producing results that closely match the gold standard technique (Shung et al., 2020; Hupel and Stütz, 2022). While achieving 100% accuracy is rare, if it occurs, the test may be appropriate for diagnostic confirmation to support critical decision-making (Ahmad et al., 2022; Rashid et al., 2024). Importantly, this guideline promotes decision-making based on performance ranges rather than rigid numerical cut-offs. In addition, statistical decision should be made guided by wisdom rather than focussing on the interpretation of *p*-value (Bujang, 2025). To facilitate implementation, the accompanying checklist as presented in Table 3 summarizes the key decision points.

**Table 3:** Checklist for cut-off determination based on AUC analysis.

No. steps
1. Assess AUC value. 2. Clarify scientific purpose of test: i.e., Screening, triage, or definitive diagnosis. 3. Evaluate misclassification risks: ° Set priority (i.e., Is a false negative or false positive more harmful?) 4. Choose selection strategy accordingly (Refer Table 2): ° Optimal solution (i.e., Youden's Index or other techniques) ° 100% sensitivity (when missing a case is dangerous) ° 100% specificity (when false positives have high cost) ° Screening strategy either emphasize on sensitivity or specificity 5. Document justification of selected cut-off: Include rationale based on AUC, scientific context, and statistical performance. 6. Based on selected cut off(s), report sensitivity, specificity, and other indicators whenever is necessary (i.e., 95% CIs, negative predictive value, positive predictive value) to ensure transparency and scientific evidence.

The checklist presented in Table 2 did not incorporate cut-off values for Positive Predictive Value (PPV) or Negative Predictive Value (NPV). The Area Under the Curve (AUC) reflects a test's overall discriminative ability, showing how effectively it can differentiate between individuals with and without a condition across all possible thresholds. In contrast, PPV and NPV indicate the probability that a person who tests positive truly has the condition, or that a person who tests negative truly does not have the condition, respectively. Both of these measures are influenced not only by the chosen cut-off point but also by the prevalence of the condition in the population. Consequently, researchers may adjust or revise these cut-offs repeatedly to achieve a balance that produces satisfactory predictive performance for the specific study population and research objectives.

Building upon these considerations, determining an appropriate cut-off score extends beyond maximizing statistical indicators and requires careful consideration of the broader clinical and public health context. To ensure a high-value and high-quality screening program, clinical decision-makers should account for disease prevalence and the potential burden of false-positive results when establishing acceptable specificity thresholds. In populations with low disease prevalence, even modest false-positive rates may generate substantially more false-positive than true-positive results, potentially reducing the Positive Predictive Value (PPV) and increasing unnecessary clinical burden. Consequently, screening programs targeting rare conditions often seek very high specificity, sometimes exceeding 99%, to maintain acceptable predictive performance. Higher specificity becomes particularly important when positive screening results lead to invasive, risky, or expensive follow-up procedures, as reducing false-positive findings may help minimize unnecessary interventions, patient anxiety, and healthcare costs. Nevertheless, prioritizing specificity should be balanced against sensitivity to avoid overlooking true cases and compromising early detection efforts.

## 7 Conclusion

Cut-off score selection remains a critical yet under-discussed element in screening/diagnostic research. This review paper offers a structured, AUC-based guideline that integrates statistical measures with reasonable scientific decision-making. By providing stratified recommendations and a practical checklist, the framework empowers researchers and clinicians to select thresholds that are both statistically sound and scientifically meaningful. Adoption of this approach can improve the consistency, transparency, and utility of diagnostic test reporting, ultimately enhancing the translation of diagnostic tools into practice.

## Data Availability

The original contributions presented in the study are included in the article/supplementary material, further inquiries can be directed to the corresponding author.

## References

[B1] Ahmad G. N. Fatima H. Abbas M. Rahman O. Alqahtani M. S. (2022). Mixed machine learning approach for efficient prediction of human heart disease by identifying the numerical and categorical features. Appl. Sci. 12:7449. doi: 10.3390/app12157449

[B2] Bossuyt P. M. Reitsma J. B. Bruns D. E. Gatsonis C. A. Glasziou P. P. Irwig L. . (2015). STARD 2015: an updated list of essential items for reporting diagnostic accuracy studies. BMJ 351:h5527. doi: 10.1136/bmj.h5527 26511519 PMC4623764

[B3] Bujang M. A. (2023). An elaboration on sample size planning for performing a one-sample sensitivity and specificity analysis by basing on calculations on a specified 95% confidence interval width. Diagnostics 13:1390. doi: 10.3390/diagnostics13081390 37189491 PMC10137289

[B4] Bujang M. A. (2025). The dilemma and wisdom in translating p values: a collaborative approach to strengthening scientific validity. BioMed Res. Int. 2025:6703756. doi: 10.1155/bmri/6703756 40201408 PMC11976038

[B5] Bujang M. A. (2026). A power primer revisited. Ind. J. Psychol. Med. 48, 206–208. doi: 10.1177/02537176261421310 PMC12880927 41659197

[B6] Bujang M. A. Adnan T. H. (2016). Requirements for minimum sample size for sensitivity and specificity analysis. J. Clin. Diagn. Res. 10, YE01–YE06. doi: 10.7860/JCDR/2016/18129.8744 PMC5121784 27891446

[B7] Bujang M. A. Kuan P. X. Sapri F. E. Liu W. J. Musa R. (2019). Risk factors for 3-year mortality and a tool to screen patients in the dialysis population. Ind. J. Nephrol. 29, 235–241. doi: 10.4103/ijn.IJN_152_18 PMC6668314 31423056

[B8] Bujang M. A. Kuan P. X. Tiong X. T. Saperi F. E. Ismail M. Mustafa F. I. . (2018). The all-cause mortality and a screening tool to determine high-risk patients among prevalent type 2 diabetes mellitus patients. J. Diab. Res. 2018:4638327. doi: 10.1155/2018/4638327 PMC6079498 30116741

[B9] Cohen J. (1992). A power primer. Psychol. Bull. 112, 155–159. doi: 10.1037/0033-2909.112.1.155 19565683

[B10] Eisenberg M. J. (1995). Accuracy and predictive values in clinical decision-making. Cleveland Clin. J. Med. 62, 311–316. doi: 10.3949/ccjm.62.5.311 7586487

[B11] Fluss R. Faraggi D. Reiser B. (2005). Estimation of the Youden index and its associated cutoff point. Biometr. J. 47, 458–472. doi: 10.1002/bimj.200410135 16161804

[B12] Gilat G. G. Linn S. (2018). Detection and diagnostic overall accuracy measures of medical tests; *Rambam Maimon. Med. J*. 9:e0027. doi: 10.5041/RMMJ.10351 PMC6186004 30309437

[B13] Greiner M. Pfeiffer D. Smith R. D. (2000). Principles and practical application of the receiver-operating characteristic analysis for diagnostic tests. Prevent. Vet. Med. 45, 23–41. doi: 10.1016/S0167-5877(00)00115-X 10802332

[B14] Habibzadeh F. Habibzadeh P. Yadollahie M. (2016). On determining the most appropriate test cut-off value: the case of tests with continuous results. Biochem. Medica 26, 297–307. doi: 10.11613/BM.2016.034 PMC5082211 27812299

[B15] Hajian-Tilaki K. (2018). The choice of methods in determining the optimal cut-off value for quantitative diagnostic test evaluation. Stat. Methods Med. Res. 27, 2374–2383. doi: 10.1177/0962280216680383 28673124

[B16] Hassanzad M. Hajian-Tilaki K. (2024). Methods of determining optimal cut-point of diagnostic biomarkers with application of clinical data in ROC analysis: an update review. BMC Med. Res. Methodol. 24:84. doi: 10.1186/s12874-024-02198-2 38589814 PMC11000303

[B17] Hupel T. Stütz P. (2022). Adopting hyperspectral anomaly detection for near real-time camouflage detection in multispectral imagery. Remote Sens. 14:3755. doi: 10.3390/rs14153755

[B18] Kumar Y. Koul A. Singla R. Ijaz M. F. (2023). Artificial intelligence in disease diagnosis: a systematic literature review, synthesizing framework and future research agenda. J. Amb. Intell. Human. Comput. 14, 8459–8486. doi: 10.1007/s12652-021-03612-z PMC8754556 35039756

[B19] Kwegyir-Aggrey K. Gerchick M. Mohan M. Horowitz A. Venkatasubramanian S. (2023). “The misuse of AUC: what high impact risk assessment gets wrong,” in Proceedings of the 2023 ACM Conference on Fairness, Accountability, and Transparency (New York, NY: ACM), 1570–1583. doi: 10.1145/3593013.3594100

[B20] Liu X. (2012). Classification accuracy and cut point selection. Stat. Med. 31, 2676–2686. doi: 10.1002/sim.4509 22307964

[B21] Lobo J. M. Jiménez-Valverde A. Real R. (2008). AUC: a misleading measure of the performance of predictive distribution models. Glob. Ecol. Biogeogr. 17, 145–151. doi: 10.1111/j.1466-8238.2007.00358.x

[B22] López-Ratón M. Rodríguez-Álvarez M. X. Cadarso-Suárez C. Gude-Sampedro F. (2014). OptimalCutpoints: an R package for selecting optimal cutpoints in diagnostic tests. J. Stat. Softw. 61, 1–36. doi: 10.18637/jss.v061.i08

[B23] Md Sani S. S. Han W. H. Bujang M. A. Ding H. J. Ng K. L. Amir Shariffuddin M. A. (2017). Evaluation of creatine kinase and liver enzymes in identification of severe dengue. BMC Infect. Dis. 17:505. doi: 10.1186/s12879-017-2601-8 28732476 PMC5520296

[B24] Metz C. E. (1978). Basic principles of ROC analysis. Semin. Nucl. Med. 8, 283–298. doi: 10.1016/S0001-2998(78)80014-2 112681

[B25] Nassif A. B. Talib M. A. Nasir Q. Afadar Y. Elgendy O. (2022). Breast cancer detection using artificial intelligence techniques: a systematic literature review. Artif. Intell. Med. 127:102276. doi: 10.1016/j.artmed.2022.102276 35430037

[B26] Pepe M. S. (2003). The Statistical Evaluation of Medical Tests For Classification and Prediction. Oxford: Oxford University Press. doi: 10.1093/oso/9780198509844.001.0001

[B27] Perkins N. J. Schisterman E. F. (2006). The inconsistency of “optimal” cut-points obtained using two criteria based on the receiver operating characteristic curve. Am. J. Epidemiol. 163, 670–675. doi: 10.1093/aje/kwj063 16410346 PMC1444894

[B28] Rashid J. Qaisar B. S. Faheem M. Akram A. Amin R. U. Hamid M. (2024). Mouth and oral disease classification using InceptionResNetV2 method. Multimedia Tools Appl. 83, 33903–33921. doi: 10.1007/s11042-023-16776-x

[B29] Rota M. Antolini L. (2014). Finding the optimal cut-point for Gaussian and gamma distributed biomarkers. Comput. Stat. Data Anal. 69, 1–4. doi: 10.1016/j.csda.2013.07.015

[B30] Samsudin A. Yap Yon Lek M. D. Loh Yoon Doong M. B. Chit H. H. Adam M. Bujang P. H. (2022). Palliative prognostic index as a predictor of mortality among geriatric patients with advanced chronic medical conditions. Med. J. Malaysia 77, 468–474. 35902937

[B31] Schisterman E. F. Perkins N. J. Liu A. Bondell H. (2008). Optimal cut-point and its corresponding Youden index to discriminate individuals using pooled blood samples. Epidemiology 19, 73–81. doi: 10.1097/01.ede.0000147512.81966.ba 15613948

[B32] Senthil M. P. Salowi M. A. Bujang M. A. Kueh A. Siew C. M. Sumugam K. . (2015). Risk factors and prediction models for retinopathy of prematurity. Malays. J. Med. Sci. 22, 57–63. PMC5295742 28239269

[B33] Shung D. L. Au B. Taylor R. A. Tay J. K. Laursen S. B. Stanley A. J. . (2020). Validation of a machine learning model that outperforms clinical risk scoring systems for upper gastrointestinal bleeding. Gastroenterology 158, 160–167. doi: 10.1053/j.gastro.2019.09.009 31562847 PMC7004228

[B34] Sounderajah V Ashrafian H. Golub R. M. Shetty S. De Fauw J. Hooft L. (2021). Developing a reporting guideline for artificial intelligence-centred diagnostic test accuracy studies: The STARD-AI protocol. BMJ Open 11:e047709. doi: 10.1136/bmjopen-2020-047709 PMC8240576 34183345

[B35] Tan S. M. Loh S. F. Bujang M. A. Haniff J. Abd Rahman F. N. Ismail F. . (2013). Validation of the malay version of children's depression inventory. Int. Med. J. 20, 177–182.

[B36] Tiong X. T. Abdullah N. S. S. Bujang M. A. Joon C. K. Wee H. L. Fong Y. . (2018). Validation of the Kessler's psychological distress scale (K10 and K6) in a Malaysian population. ASEAN J. Psych. 19, 1–9.

[B37] Unal I. (2017). Defining an optimal cut-point value in ROC analysis: an alternative approach. Comput. Math. Methods Med. 2017:3762651. doi: 10.1155/2017/3762651 28642804 PMC5470053

[B38] Vaish R. Dwivedi U. D. Tewari S. Tripathi S. M. (2021). Machine learning applications in power system fault diagnosis: research advancements and perspectives. Eng. Appl. Artif. Intell. 106:104504. doi: 10.1016/j.engappai.2021.104504

[B39] Verbakel J. Y. Steyerberg E. W. Uno H. De Cock B. Wynants L. Collins G. S. . (2020). ROC curves for clinical prediction models part 1: ROC plots showed no added value above the AUC when evaluating the performance of clinical prediction models. J. Clinic. Epidemiol. 126, 207–216. doi: 10.1016/j.jclinepi.2020.01.028 32712176

[B40] Vickers A. J. Elkin E. B. (2006). Decision curve analysis: a novel method for evaluating prediction models. Med. Decis. Mak. 26, 565–574. doi: 10.1177/0272989X06295361 PMC2577036 17099194

[B41] Youden W. J. (1950). Index for rating diagnostic tests. Cancer 3, 32–35. doi: 10.1002/1097-0142(1950)3:1<32::aid-cncr2820030106>3.0.co;2-3 15405679

